# Calcineurin Inhibitors Synergize with Manogepix to Kill Diverse Human Fungal Pathogens

**DOI:** 10.3390/jof8101102

**Published:** 2022-10-19

**Authors:** Sean D. Liston, Luke Whitesell, Mili Kapoor, Karen J. Shaw, Leah E. Cowen

**Affiliations:** 1 Department of Molecular Genetics, University of Toronto, Toronto, ON M5G 1M1, Canada; 2 Pfizer Inc., San Diego, CA 92121, USA; 3 Hearts Consulting Group, LLC, Poway, CA 92064, USA

**Keywords:** fosmanogepix, manogepix, FMGX, MGX, APX001, APX001A, Gwt1, FK506, antifungal, GPI anchor, glycosylphosphatidylinositol

## Abstract

Invasive fungal infections have mortality rates of 30–90%, depending on patient co-morbidities and the causative pathogen. The frequent emergence of drug resistance reduces the efficacy of currently approved treatment options, highlighting an urgent need for antifungals with new modes of action. Addressing this need, fosmanogepix (*N*-phosphonooxymethylene prodrug of manogepix; MGX) is the first in a new class of gepix drugs, and acts as a broad-spectrum, orally bioavailable inhibitor of the essential fungal glycosylphosphatidylinositol (GPI) acyltransferase Gwt1. MGX inhibits the growth of diverse fungal pathogens and causes accumulation of immature GPI-anchored proteins in the fungal endoplasmic reticulum. Relevant to the ongoing clinical development of fosmanogepix, we report a synergistic, fungicidal interaction between MGX and inhibitors of the protein phosphatase calcineurin against important human fungal pathogens. To investigate this synergy further, we evaluated a library of 124 conditional expression mutants covering 95% of the genes encoding proteins involved in GPI-anchor biosynthesis or proteins predicted to be GPI-anchored. Strong negative chemical-genetic interactions between the calcineurin inhibitor FK506 and eleven GPI-anchor biosynthesis genes were identified, indicating that calcineurin signalling is required for fungal tolerance to not only MGX, but to inhibition of the GPI-anchor biosynthesis pathway more broadly. Depletion of these GPI-anchor biosynthesis genes, like MGX treatment, also exposed fungal cell wall (1→3)-β-D-glucans. Taken together, these findings suggest the increased risk of invasive fungal infections associated with use of calcineurin inhibitors as immunosuppressants may be mitigated by their synergistic fungicidal interaction with (fos)manogepix and its ability to enhance exposure of immunostimulatory glucans.

## 1. Introduction

Fungi, predominately from the genera *Aspergillus, Cryptococcus*, and *Candida*, cause invasive infections that kill ~1.5 million people annually [1,2]. Risk groups for these infections include individuals in the developing world with poorly controlled HIV infection and in the developed world, recipients of cytotoxic chemotherapeutics and immunosuppressive therapies such as corticosteroids and calcineurin inhibitors [3,4,5]. Despite best-available antifungal treatments, mortality rates for invasive fungal infections remain disturbingly high at 30–90%, depending on the particular co-morbidities complicating management and the specific fungal pathogen involved [6]. Approved treatments rely on only three major classes of antifungal drugs, which target ergosterol in the fungal cell membrane or synthesis of (1→3)-β-D-glucans in the fungal cell wall. These drugs have important limitations with regard to host toxicity/tolerability and/or restricted spectrum of activity which limit their efficacy. Further, drug-resistant fungi have emerged in the clinic, some of which are resistant to all three drug classes [7,8,9,10]. Therefore, an urgent, unmet need exists for new broad-spectrum antifungal drugs, ideally with non-overlapping mechanisms of action to current drug classes [11].

Inhibiting glycosylphosphatidylinositol (GPI)-anchoring of proteins appears to offer a promising new strategy to treat invasive fungal infections [12,13,14,15]. GPI anchors are conserved glycolipids that are assembled and post-translationally attached to proteins in the eukaryotic endoplasmic reticulum (ER) [16]. GPI anchors are essential for trafficking and tethering otherwise soluble proteins to the cell surface. Blocking the GPI anchoring of proteins is particularly toxic to fungi because GPI-anchored proteins are integral to fungal cell wall architecture. A number of small molecules targeting GPI biosynthesis enzymes are in development as antifungal drugs, which benefit from further specificity due to divergence between GPI-biosynthesis proteins in fungi and mammals [17,18,19]. The most advanced of these agents, fosmanogepix (FMGX), is a phosphonooxymethylene prodrug of manogepix (MGX; formerly APX001A, E1210), which inhibits Gwt1, an acyl-CoA-dependent glucosaminyl-phosphatidyl inositol (GlcN-PI) acyltransferase that is essential for biosynthesis of GPI anchors [20,21,22]. FMGX has been well tolerated in human clinical trials to date and its metabolite MGX has demonstrated broad-spectrum antifungal activity against the major fungal pathogens *Aspergillus fumigatus*, *Cryptococcus neoformans*, and *Candida albicans* [14]. A close structural analog of MGX with up to 32-fold improved potency against *C. neoformans* (APX2039) has also been reported [23].

GPI anchor biosynthesis inhibitors have pleotropic effects in part due to the diverse functions of GPI-anchored proteins. Genetic findings indicate that complete loss of *GWT1* function is fungicidal, while partial blockade of the pathway by inhibitors is fungistatic [19,20]. In *C. albicans*, MGX inhibits the transition from yeast to filamentous growth forms, a key virulence factor for this pathogen [24,25]. MGX treatment also reduces adherence to surfaces and biofilm formation [20]. This effect is not surprising because many GPI-anchored proteins mediate interactions with biotic and abiotic surfaces, including adhesins of the agglutinin-like sequence (Als)-family which require modification with a GPI anchor for function [20,26]. Chemically distinct Gwt1 inhibitors have also been reported to alter cell wall architecture and uncover (1→3)-β-D-glucan at the fungal cell surface, which activates macrophages and is predicted to enhance clearance by immune cells in the host [19,27].

One of the fundamental effects of Gwt1 inhibition is accumulation of immature GPI proteins in the fungal ER, which induces the cytoprotective Unfolded Protein Response (UPR). A key component of the UPR in fungi is activation of the calcium/calmodulin-dependent protein phosphatase calcineurin [28,29]. Here, inspired by previous evidence that induction of the UPR protects *C. albicans* from the toxic effects of Gwt1 inhibition [19,30], we examined the effect of inhibiting calcineurin on the antifungal activity of MGX. Discovering synergistic, fungicidal interaction between MGX and impairment of calcineurin, we used a chemogenomic approach to screen the GPI-anchor system more broadly for negative chemical-genetic interactions with FK506, a classical calcineurin inhibitor. Eleven such GPI-anchor biosynthesis genes were identified. Depletion of most of these genes, like MGX treatment, also exposed fungal cell wall (1→3)-β-D-glucans. Taken together, these findings suggest that rather than impairing the antifungal activity of MGX after administration of fosmanogepix, the concomitant administration of FK506, as might be expected to occur frequently in hematopoietic and solid organ transplant settings, has the striking potential to enhance the efficacy of MGX.

## 2. Materials and Methods

### 2.1. Fungal Strains Used

*C. albicans*:

ATCC 90,028 (Clinical isolate) [31]

SN95 (*arg4/arg4, his1/his1, URA3/ura3::imm434, IRO1/iro1::imm434*) [32]

CaLC909 (As SN95, *cna1::FRT/cna1::FRT*) [33]

CaSS1 (*his3::hisG/his3::hisG, leu2::tetR-GAL4AD-URA3/LEU2*) [34]

*C. neoformans*:

KN99a (Clinical isolate) [35]

*A. fumigatus*:

Af293 (Clinical isolate) [36]

### 2.2. Culture Media and Conditions

Fungal strains were routinely grown in Yeast Extract Peptone Dextrose (YPD) medium (1% yeast extract, 2% peptone, 2% glucose), from cryopreserved cultures stored at −80 °C in 25% glycerol. Cyclosporin A (CsA; Cayman Chemical Company 12088-100), FK506 (Invivogen, tlrl-fk5), MGX (Amplyx Pharmaceuticals Inc.), and APX2039 (Amplyx Pharmaceuticals Inc.) stocks were prepared in DMSO at 10 mM. Chemical structures are summarized in Figure S1. Doxycycline hydrochloride (DOX; Bio Basic Canada) and propidium iodide (Millipore Sigma) stocks were prepared at 50 mg/mL in water and filter sterilized.

### 2.3. Dose-Response Assays

Cultures were colony purified on YPD agar prior to use in experiments. Overnight cultures were prepared in 2 mL YPD and incubated at 30 °C for ~16 h, until cultures reached saturation. Cultures were diluted to 1 × 10^4^ cells/mL in fresh medium, then dispensed at 200 µL/well into 96-well plates. Compounds were added to wells using a D300e Drug Dispensor (Tecan) to indicated final concentrations. Cultures were statically incubated for 24–72 h at 30 °C. Optical densities were determined by spectrophotometry (SpectraMax M2, Molecular Devices). For metabolic activity assays, growth was assessed by addition of 20 µL of AlamarBlue (Invitrogen) diluted 1:4 in fresh growth medium. Cultures were mixed by orbital shaking for 1 min, then incubated for 3 h at 30 °C. Alamar Blue fluorescence was then measured using a Spark multimode plate reader (Tecan) at 535(25)/595(5) nm (Excitation/Emission). Background optical densities/fluorescence from medium in mock inoculated wells was subtracted.

### 2.4. Bioinformatic Analyses

Open reading frame (ORF) translations for *C. albicans* SC5314 (Assembly 22) were downloaded from the Candida Genome Database [37]. Predicted proteins containing an N-terminal signal peptide were identified using SignalP 4.0 [38]. Predicted GPI proteins were identified from this set using PredGPI [39]. GPI anchor biosynthesis enzymes were identified based on homology to those identified in *S. cerevisiae* [16,40]. Conditional expression strains for GPI anchor biosynthesis and predicted GPI-anchored proteins were constructed for the GRACE library of *C. albicans* [34,41]. Strains from the GRACE collection included in this work are summarized in Table S1.

### 2.5. Chemical Genetic Interaction Screen

Triplicate 96-well plates containing 100 µL YPD medium were inoculated with *C. albicans* and mutant derivates and incubated overnight at 30 °C. Sub-culture was performed at ~1:100 using a 96-well replicator into 96-well plates containing 100 µL YPD medium or YPD medium supplemented with 0.05 µg/mL DOX and then incubated overnight at 30 °C. Cultures were then sub-cultured at ~1:100 using a 96-well replicator into 96-well plates containing 100 µL YPD medium or YPD medium supplemented with DOX (0.05 µg/mL), FK506 (0.1 µg/mL), or the combination of DOX + FK506. Cultures were incubated at 30 °C for 24 h, then growth was measured by OD_600_. Background optical densities from medium in mock inoculated wells was subtracted, and growth was normalized to drug-free wild-type cultures. Chemical genetic interaction scores were calculated using a multiplicative model, such that the relative growth in FK506 and DOX was subtracted from the product of relative growth in FK506 and relative growth in DOX [42]. Statistical comparisons were performed, and figure panels were prepared in GraphPad Prism (v. 9.4.0).

### 2.6. Cell Wall Staining and Quantification

To evaluate the effect of MGX on WT *C. albicans*, cells were cultured in YPD supplemented with various concentrations of the compound for 24 h at 30 °C. Cells were then fixed by addition of formaldehyde to 2% (*v/v*) final and incubated for 24 h at 4 °C prior to staining. To evaluate GRACE strains, 96-well plates containing 100 µL YPD medium were inoculated with parental *C. albicans* and mutant derivatives and incubated overnight at 30 °C. Subcultures were performed at ~1:100 using a 96-well replicator into 100 µL YPD medium or YPD medium supplemented with 0.05 µg/mL DOX and then incubated overnight at 30 °C. The next day, subculture was again performed at ~1:100 using a 96-well replicator into 100 µL YPD medium or YPD medium supplemented with 5 µg/mL DOX. These subcultures were then incubated for 4 h at 30 °C. For validation experiments, subcultures were performed at 1:100 into 2 mL YPD medium or YPD medium supplemented with 5 µg/mL DOX and incubated at 30 °C with 200 rpm shaking for 4 h. Cells were then fixed by addition of formaldehyde to 2% (*v/v*) final and incubated for 24 h at 4 °C prior to staining.

For staining, fixed cell suspensions were transferred to Millipore DV filter plates for chitin or glucan staining. Washes were performed in filter plates by centrifugation at 1000× *g* for 1 min at 4 °C. For glucan staining, cells were washed 3× with 100 µL phosphate buffered saline (PBS), then blocked with 100 µL PBS containing 3% (*w/v*) bovine serum albumin (PBS-BSA) for 15 min. Cells were immunostained by addition of 100 µL anti (1→3)-β-D-glucan monoclonal antibody (Biosupplies Australia) diluted at 1:500 in PBS-BSA containing and then incubated at RT for 1 h. Cells were washed 3× with 100 µL PBS supplemented with 0.05% (*v/v*) Tween 20 (PBS-T). Cells were then incubated with 100 µL goat anti-mouse antibody conjugated to AlexaFluor488 (Invitrogen A21121) diluted at 1:500 in PBS-BSA at RT for 1 h. Cells were washed 3× with 100 µL PBS-T then resuspended in 100 µL PBS-BSA for analysis by flow cytometry and fluorescence microscopy.

For calcofluor white (CFW) staining, 100 µL of PBS-BSA containing 2 µg/mL CFW was added to 100 µL of fixed cultures and incubated at RT for 1 h in the dark. Cells were then washed 3× with 100 µL PBS-T and resuspended in 100 µL PBS-BSA for analysis by flow cytometry and fluorescence microscopy.

### 2.7. Flow Cytometry

Flow cytometry was performed on a CytoFLEX flow cytometer (Beckman Coulter) controlled by CytExpert software (Beckman Coulter, version 2.4). Forty thousand events were collected for each sample, which were gated to exclude debris smaller than yeast cells. Median fluorescence intensities are reported for populations representing > 95% of total events. To construct boxplots, selected gated populations were exported from CytExpert as fcs3 files. Files were analyzed in R v4.2.1 (https://www.R-project.org/, accessed on 28 July 2022) using the FlowCore package v2.9.1 (https://www.bioconductor.org/packages/release/bioc/manuals/flowCore/man/flowCore.pdf, accessed on 28 July 2022).

### 2.8. Microscopy

Differential interference contrast (DIC) and fluorescence micrographs were collected on an AxioImager.M1 (Zeiss) using ET HQ DsRed and EGFP filter sets (Chroma Technology Corp). Images were acquired for identical exposure times and processed using Zen Pro (Zeiss).

## 3. Results

### 3.1. Gwt1 Inhibitors Synergize with Classical Immunosuppressive Calcineurin Inhibitors

**Figure 1:**
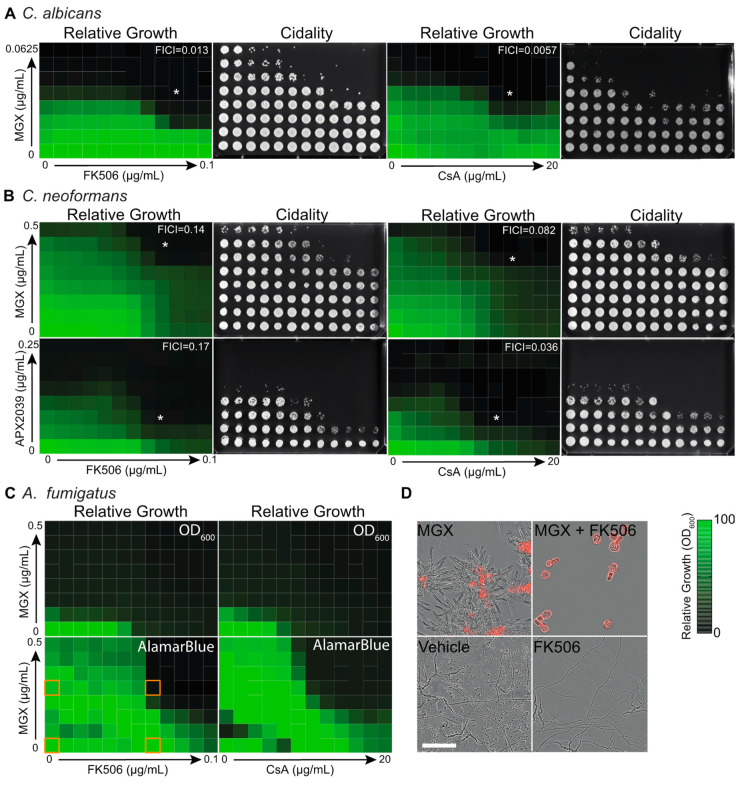
Gwt1 inhibitors synergize with calcineurin inhibitors to kill diverse human fungal pathogens. (**A**) FK506 and CsA synergize with MGX to inhibit growth of *C. albicans*. Two-fold dose response matrices for MGX and FK506 or MGX and CsA tested against *C. albicans*. Growth was determined by OD600 after 24 h incubation in YPD medium and normalized to vehicle control wells. Data are mean relative growth for three replicate wells from a representative experiment. The lowest FICI value is inset (white text), and the corresponding well marked with an asterisk. To assess viability after combination drug treatments, cells were transferred onto drug-free YPD agar and photographed after culture for an additional 48 h. A scale bar for all heat-map depictions presented in panels (**A**–**C**) is provided to the right of panel (**D**). (**B**) FK506 and CsA synergistically interact with MGX or the structural analog APX2039 to inhibit growth of *C. neoformans*. Assays were performed as in (**A**), except optical densities were determined after 48 h growth. (**C**) FK506 and CsA synergistically interact with MGX to inhibit growth of *A. fumigatus*. Assays were performed as in (**A**), except optical densities were determined at 48 h. Relative metabolic activity was assessed by measuring Alamar blue dye reduction. Data are mean relative fluorescence intensity normalized to vehicle control wells for three replicate wells from a representative experiment. (**D**) Merged phase contrast and fluorescence photomicrographs visualizing the dose-response assay wells outlined in orange in panel (**C**) after addition of propidium iodide (1 µg/mL) to identify dead cells. Scale bar,100 µm. Experiments were repeated at least two times with similar results.

To begin evaluating the role of calcineurin in responding to inhibition of GPI anchor biosynthesis across the most common human fungal pathogens, we performed checkerboard dose-response assays with MGX and the clinically relevant calcineurin inhibitors FK506 and cyclosporin A (CsA). against clinical isolates of *C. albicans* (ATCC90028), *C. neoformans* (KN99a) and *A. fumigatus* (Af293). Both of these widely used immunosuppressants strongly synergized with MGX against *C. albicans*, yielding fractional inhibitory concentration indices (FICI) below 0.5 (Figure 1A).

To better define the dose-response landscape for this interaction, Bliss synergy analysis was performed on the MGX-FK506 dataset (Figure S2). Robust synergy (Bliss score 13.97) was seen across the landscape with a value of 40.5 calculated for the most synergistic area [43]. Of significance from the therapeutic perspective, combination exposure also converted the fungistatic activity of the individual agents to fungicidal. Against a reference isolate of *C. neoformans* (KN99a), combination drug exposure also provided synergistic fungicidal activity (Figure 1B). For this organism, we assessed both MGX and APX2039, a closely related structural analog of MGX with selectively improved activity against *Cryptococcus* spp [23]. Unlike MGX, APX2039 demonstrated single agent fungicidal activity against *C. neoformans* at the concentrations tested. Perhaps its 32-fold greater potency permitted near-complete target inhibition, which based on gene-deletion data in *C. albicans* would be lethal [44]. As endpoints for monitoring antifungal activity against the mold *A. fumigatus*, we used both optical density and metabolic activity to monitor growth, and cidality was assessed by microscopy using propidium iodide dye exclusion as an endpoint to avoid potential artifacts arising from the filamentous morphology of this organism (Figure 1C,D).

### 3.2. Chemical-Genetic Interactions Confirm Target-Based Synergy

**Figure 2:**
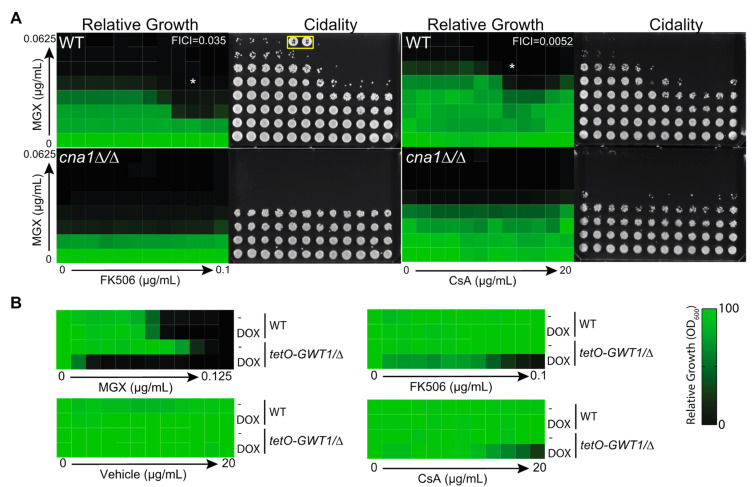
Calcineurin and Gwt1 are responsible for synergistic interaction between MGX and FK506 or CsA. (**A**) Dose-response matrices were generated for MGX and FK506 or MGX and Cyclosporin A against wild-type *C. albicans* and a *cna1Δ/Δ* mutant derivative. Growth was monitored by OD_600_ after 24 h incubation in YPD medium and normalized to vehicle control wells. Data are mean relative growth for triplicate wells from a representative experiment. This experiment was performed in two additional replicates with similar results. To assess viability after combination treatments, cells were transferred onto drug-free YPD agar and photographed after 48 h growth. Contaminated wells are marked with a pound sign and surrounded with a yellow box. The lowest FICI value is inset (white text), and the corresponding well indicated with an asterisk. (**B**) Transcriptional repression of GWT1 sensitizes *C. albicans* to FK506 and CsA. Dose response assays were performed with *C. albicans* or tetO-GWT1/Δ mutant derivative in the presence or absence of DOX. Growth was monitored by OD_600_ after 24 h incubation and normalized to vehicle control wells. Data are mean relative growth for three replicate wells from a representative experiment. This experiment was performed in two additional replicates with similar results.

Given that two structurally distinct immunosuppressants synergized with Gwt1 inhibitors against a broad spectrum of fungi, we next sought to confirm that disruption of calcineurin and Gwt1 function, rather than potential off-target effects of the compounds, were responsible for the synergy demonstrated in checkerboard dose-response tests. To complement pharmacological data, we used molecular genetic techniques to disrupt the function of calcineurin. Homozygous deletion of *CNA1,* the gene encoding the catalytic subunit of calcineurin, markedly sensitized *C. albicans* to MGX and rendered the compound fungicidal (Figure 2A). In addition, the *cna1* mutant was not further sensitized to MGX by exposure to either CsA or FK506, confirming that these compounds act via calcineurin inhibition to increase rather than some other mechanism.

To confirm that sensitization to MGX by the immunosuppressants was due to inhibition of Gwt1 rather than some other target, we used a previously published Gene Replacement and Conditional Expression (GRACE) strain in which the native promoter of one allele of *GWT1* was replaced with a tetracycline-repressible promoter (*tetO*) and the other allele was deleted (*tetO-GWT1/Δ*) [44]. In the absence of repression by doxycycline (DOX, a tetracycline analog), *GWT1* expression from the strong *tetO* promoter is expected to be greater than expression in the wild-type parental strain [30,41]. Consistent with this expectation, the mutant strain demonstrated reduced susceptibility to MGX in the absence of DOX (Figure 2B). In the presence of a low DOX concentration, it showed marked sensitization to MGX consistent with the principle of reduced gene-dosage conferring hypersensitivity to an inhibitor of the gene’s protein product (Figure 2B). Having confirmed appropriate sensitization to MGX in our DOX-regulated strain, we then measured the effect of down-regulating *GWT1* expression on concentration-dependent growth inhibition by immunosuppressants and confirmed DOX-dependent sensitization to both FK506 and CsA (Figure 2B).

### 3.3. Calcineurin Mediates Tolerance to Impairment of GPI-Anchor Biosynthesis at Multiple Steps in the Biosynthetic Pathway

**Figure 3:**
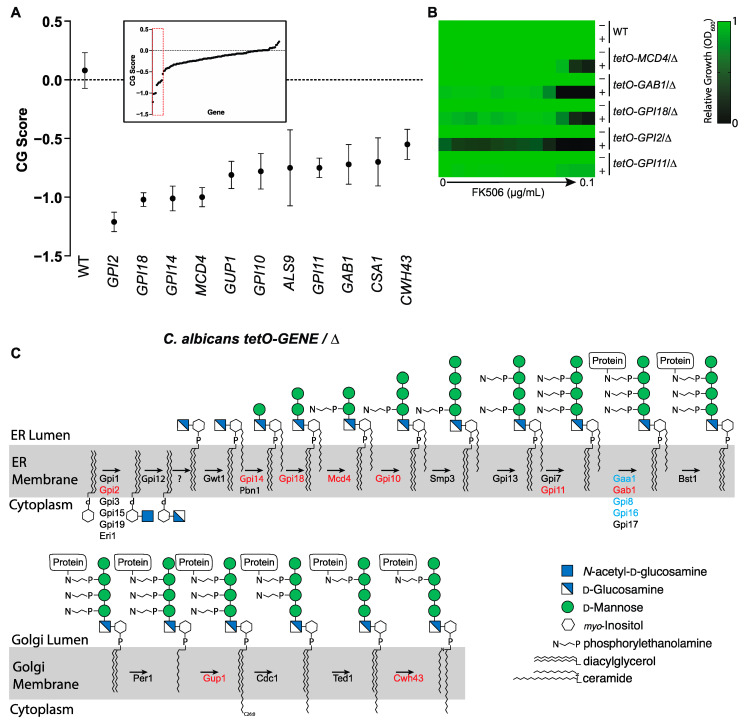
Transcriptional repression of diverse GPI-anchor biosynthesis genes sensitizes *C. albicans* to MGX and FK506. (**A**) Screen of GPI-anchor focused library. Conditional expression strains were grown in the presence or absence of FK506 (100 ng/mL) to inhibit calcineurin and in the presence or absence of DOX (5 µg/mL) to repress target gene expression. Growth was assessed by OD_600_ after 24 h. Chemical genetic (CG) interaction scores are displayed as the difference between expected and observed relative growth according to a multiplicative model. Data are mean CG Score ± SEM for triplicate wells from a representative experiment. Strains with statistically significant negative CG scores are indicated (unpaired t-test, Holm–Šídák’s multiple comparison test, *p* < 0.05) are indicated. Inset depicts values obtained for entire library. Gene names based on the respective *S. cerevisiae* homologues are indicated for *C1_04280* (*GPI18*), *C_505040* (*GPI10*) and *C503120* (*GAB1*). (**B**) MIC testing confirms transcriptional repression of multiple GPI anchor biosynthesis genes confers hypersensitivity to FK506. *C. albicans* or tetO-GENE/Δ mutant derivatives were grown in YPD at 30 °C in the presence or absence of 0.05 µg/mL DOX, then sub-cultured into YPD medium ± 5 µg/mL DOX and a two-fold dilution series of FK506. Growth was determined by OD_600_ after 24 h and normalized to vehicle control wells. Data are mean relative growth for triplicate wells from a representative experiment. (**C**) Schematic depiction of GPI anchor biosynthesis. Legend depicts sugar residues of the GPI structure according to Symbol Nomenclature for Glycans [47]. Genes with CG interactions with FK506 are colored in red. Genes determined be essential based on induction of a severe growth defect in the presence of DOX are colored in blue [30,41].

To move beyond Gwt1 and identify additional calcineurin-sensitive points in GPI-anchor biosynthesis, we screened a library of 124 conditional expression mutants constructed in the same manner as the *tetO-GWT1/Δ* strain described above. This library covered 95% of the genes in *C. albicans* encoding proteins involved in GPI-anchor biosynthesis or proteins predicted to be GPI-anchored (Table S1). Assembling this set of mutants required a focused expansion of the previously published *C. albicans* Gene Replacement and Conditional Expression (GRACE) library which covered only 38 of 125 predicted GPI anchor-related genes [34,41,45]. Predictions for GPI biosynthesis proteins were made based on homology in the model yeast *Saccharomyces cerevisiae* [16] and GPI-anchored proteins were predicted by the presence of an N-terminal secretion signal and C-terminal GPI anchor motif [46]. Screening was performed in the presence or absence of DOX to identify genes for which transcriptional repression conferred hypersensitivity to FK506. Negative chemical-genetic interactions were identified for eleven genes encoding proteins involved in the GPI-anchor pathway (Figure 3A).

Interestingly, with the exception of Als9 and Csa1, individual GPI-anchored proteins were not identified as hits, suggesting that dysfunction of the overall pathway rather than failure to modify a single, specific GPI-anchored protein is most responsible for the antifungal activity of MGX. As neither *ALS9* nor *CSA1* is essential, the mechanism by which impairment of GPI-anchoring enhances toxicity upon downregulation of their expression is not clear, but worthy of future investigation. In follow-up we tested screen hits in MIC format for hypersensitivity to FK506 in the presence of DOX (Figure 3B). Results confirmed screen findings and indicated that calcineurin signaling is required to varying degrees for *C. albicans* to tolerate blockade of GPI-anchor biosynthesis at diverse points in the pathway, not just at the acylation step catalyzed by Gwt1 (Figure 3C).

### 3.4. Impairing GPI Anchor Biosynthesis at Diverse Steps Alters Cell Wall Architecture to Expose (1→3)-β-D-glucan and Increase Chitin Deposition

**Figure 4:**
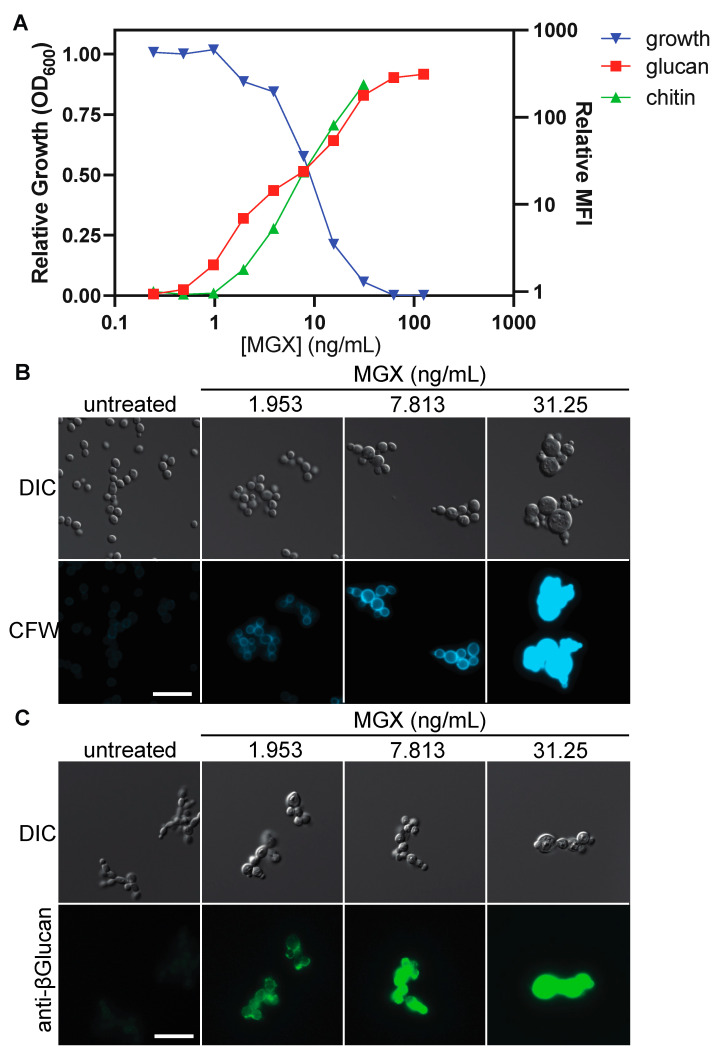
MGX treatment increases chitin levels and exposes (1→3)-β-D-glucan at the fungal cell surface. (**A**) *C. albicans* was grown in YPD supplemented with MGX for 24 h at 30 °C then growth was measured by optical density. Left: Mean relative growth for duplicate wells from a representative experiment. Cells were formaldehyde fixed, then stained with CFW or labelled with a mouse monoclonal antibody to (1→3)-β-D-glucan and goat anti-mouse secondary antibody conjugated to AlexaFluor488 and examined by flow cytometry. Right: Median fluorescence intensity for CFW and AlexaFluor488 labelled cells normalized to untreated controls (>20,000 events/sample). Fluorescence intensity for CFW staining saturated the flow cytometer at [MGX] > 31.25 ng/mL. (**B**) Representative fluorescence micrographs of CFW-stained *C. albicans* (blue) prepared as in (**A**). Scale bar, 20 µm. (**C**) Representative (1→3)-β-D-glucan immunofluorescence (green) micrographs of *C. albicans* prepared as in A. Scale bar, 20 µm.

Having found that impairment of GPI-anchor biosynthesis by MGX conferred hypersensitivity to FK506, we next asked whether it would alter fungal cell wall architecture, as previously reported for a different Gwt1 inhibitor scaffold [19]. As measured by flow cytometry of calcofluor-white (CFW) and (1→3)-β-D-glucan immunostained cells, exposure of *C. albicans* to MGX drove markedly increased cell wall chitin levels and exposed (1→3)-β-D-glucan in a concentration-dependent manner that was inversely correlated with relative fungal growth (Figure 4A). Imaging by immunofluorescence microscopy confirmed these results (Figure 4B).

**Figure 5:**
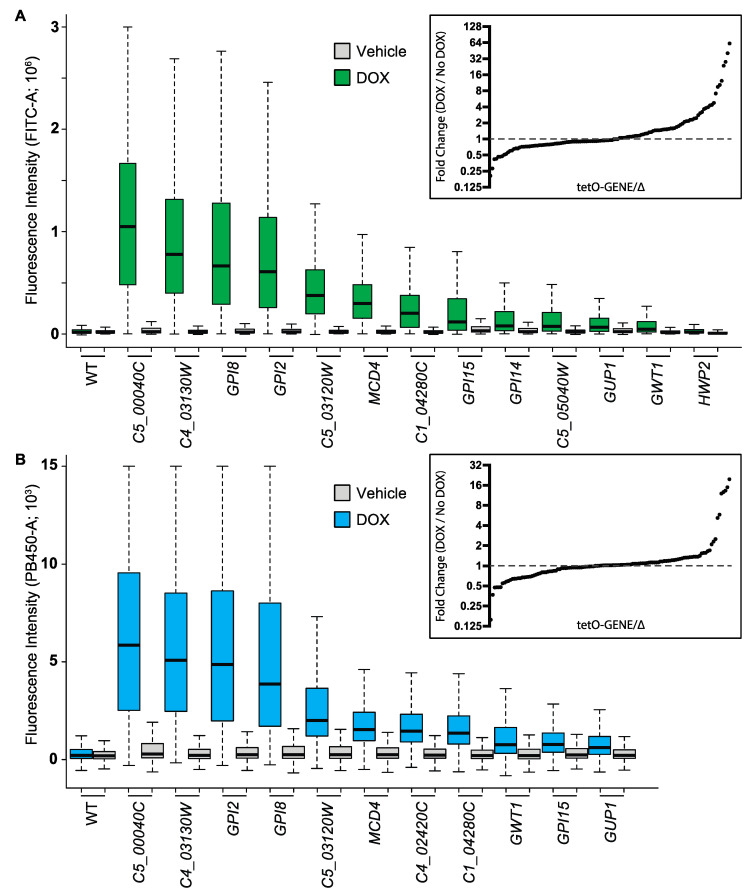
Transcriptional repression of GPI-anchor biosynthesis genes exposes (1→3)-β-D-glucan at the fungal cell surface and increases chitin levels. Conditional expression strains were grown in the presence or absence of DOX to repress target gene expression. Glucan and CFW staining and flow cytometry were performed as in Figure 4. (**A**) Glucan Staining. Median fluorescence intensity (FITC-A) is plotted for each repressible mutant in the library showing > two-fold change in median fluorescence intensity in screening and validation experiments upon repression of the indicated gene in shaking cultures. Values depicted represent a single determination (~30,000 events/sample). Inset: Screening data. Ratio of median fluorescence intensity of DOX-treated to untreated samples for all strains in the collection grown in static cultures. Values depicted represent a single determination (>1000–10,000 events/sample). (**B**) CFW Staining. Median fluorescence intensity (PB450-A) is plotted for each repressible mutant in the library showing > two-fold change in median fluorescence intensity in two experiments upon repression of the indicated gene in shaking cultures. Values depicted represent a single determination (~30,000 events/sample). Inset: Screening data. Ratio of median fluorescence intensity of DOX-treated to untreated samples for all strains in the collection grown in static culture. Values depicted represent a single determination (>1000–10,000 events/sample).

To move beyond Gwt1 and identify additional steps in GPI-anchor biosynthesis that could be required to maintain normal cell wall architecture and prevent immunostimulatory glucan exposure, we screened the GPI-focused library of 124 conditional expression mutants described above. A greater than two-fold increase in staining for surface glucan exposure was seen for repression of 13 genes in the pathway (Figure 5A and Figure S3; Table S2). A greater than 2-fold increase in CFW staining for was seen for repression of 11 genes in the pathway (Figure 5B; Table S3).

Strong overlap was observed between the gene sets associated with altered glucan and CFW staining, and with the gene set previously identified based on sensitization to FK506 (Figure 3A). The most likely explanation for the absence of complete overlap is that addition of DOX achieved more complete target gene repression in strains which showed phenotypes in all assays, while in other strains in the library, repression was more modest (false negatives). Taken together, these data suggest that in addition to Gwt1, impairment of other steps in the GPI-anchor biosynthesis pathway can also induce ER stress and disrupt fungal cell wall architecture.

## 4. Discussion

Although conserved in broad strokes from yeast to humans, the GPI-anchor biosynthesis pathway plays a unique role in fungi as GPI-anchored proteins are essential for the structural integrity of the fungal cell wall [22,48]. Within the GPI anchor pathway, the effects of impairing the acyltransferase Gwt1 have been most extensively studied [21,30]. These include accumulation of immature GPI-anchored proteins in the ER leading to cytoprotective activation of calcineurin and exposure of immunostimulatory (1→3)-β-D-glucan at the fungal cell surface [19,30]. Here, we report an important potential therapeutic implication of this basic biology by demonstrating robust synergistic interaction between the Gwt1 inhibitor MGX and the widely used calcineurin inhibitors CsA and FK506. This interaction is of more than just biological interest because as powerful inhibitors of adaptive immune function, these drugs markedly increase risk of the very same invasive fungal infections fosmanogepix is being developed to counter.

Synergistic interaction of MGX and FK506 across a wide range of clinically relevant concentrations for each drug suggests the possibility of using combination regimens to increase efficacy and reduce the emergence of antifungal resistance, especially given the cidal activity we observed for simultaneous exposure to both agents [49,50,51,52]. Further work will be needed, however, to fully define the effect of combination exposure on the magnitude and kinetics of the killing effect for different fungal species. The potential for development of target-related resistance in vitro to MGX alone has been investigated in *C. albicans*, *C. glabrata*, and *C. parapsilosis* by evaluating spontaneous resistance frequencies and was found to be relatively low, comparable to that of echinocandins [53,54]. Efflux pump-mediated mechanisms of resistance have also been reported for MGX [55]. As FK506 is known to inhibit pumps of the ABC transporter family [56], it will be interesting to see if addition of FK506, even at low concentrations that have no antifungal activity, could further reduce emergence of resistance in culture. These findings could have relevance to the use of fosmanogepix for extended periods in patients with severe fungal infections, who require concomitant immunosuppressive therapy (e.g., hematopoietic stem cell transplant setting). In these patients, the emergence of resistance would typically be favored because the duration of treatment is prolonged. In considering the implementation of such an approach, it will be essential to assess potentially problematic drug-drug interaction that could alter the pharmacology of FK506 and/or MGX. The CYP3A P450 sub-family is primarily responsible for FK506 metabolism [57]. Studies are ongoing in regards to the metabolism of fosmanogepix, but trials to date have not found significant MGX CYP3A4 inhibition.

Calcineurin inhibitors increase the risk of invasive fungal infections primarily through their compromise of adaptive immune function [58]. With this arm of the system compromised, the ability of MGX to increase glucan exposure could provide benefit beyond direct antifungal activity by enhancing recognition and destruction of fungi by phagocytes of the innate immune system [19,27,49]. In situations where therapeutic suppression of host immune function is not a goal, e.g., non-transplant settings, it may still be possible to leverage the synergistic antifungal interaction of MGX with FK506 through the use of fungal-selective, non-immunosuppressive FK506 analogues, several of which are now in preclinical development and possess single agent antifungal activity of their own [59,60]. If development succeeds, their availability would open a route to highly active combination therapy regimens, an approach which has become standard of care for life-threatening bacterial infections, HIV, tuberculosis, and malaria, but has been greatly under-explored for invasive fungal infections [11,42,52].

Based on the positive clinical experience with Gwt1 inhibitors to date, blocking GPI anchoring of proteins in fungi appears to provide an attractive antifungal strategy, but the potential of targeting other pathway components, with the exception of the mannose-ethanolamine phosphotransferase Mcd4, remains relatively under-studied [30,61]. Here, to move beyond Gwt1 and identify additional calcineurin-sensitive points in GPI-anchor biosynthesis, we screened a library of 124 conditional expression mutants covering predicted GPI biosynthesis proteins, and proteins predicted to be GPI anchored. Now covering 95% of GPI-related genes, the GPI-related mutant collection is being screened in our group for essentiality under a range of host-relevant culture conditions to identify new, potentially druggable GPI anchor-related targets for antifungal discovery and development. Beyond its translational value, however, the collection should also prove a valuable resource for the wider community and more basic studies in areas of fungal biology ranging from cell wall architecture to virulence factors and host-pathogen relationships for *C. albicans*.

## Data Availability

Raw data presented in this study are available on request from the corresponding author.

## Supplementary Materials

The following supporting information can be downloaded at: https://www.mdpi.com/article/10.3390/jof8101102/s1, Figure S1: Chemical structures of relevant compounds; Figure S2: Bliss synergy analysis of antifungal interaction between MGX and FK506; Figure S3: Transcriptional repression of GPI-anchor biosynthesis genes exposes (1→3)-β-D-glucan at the fungal cell surface; Table S1: GRACE strains used in this study. If no standard name was annotated in the Candida Genome Database, the standard name for the closest ortholog in *S. cerevisiae* is indicated in brackets; Table S2: Supplementary population statistics for Figure 5A; Table S3: Supplementary population statistics for Figure 5B.

## Abbreviations

CsA	Cyclosporin A
CFW	Calcofluor-white
DOX	Doxycycline
FICI	Fractional inhibitory concentration index
GlcN-PI	glucosaminyl-phosphatidyl inositol
GPI	glycosylphosphatidylinositol
Gwt1	GPI-anchored wall protein transfer
Mcd4	Morphogenesis checkpoint dependent 4
MGX	manogepix
FMGX	fosmanogepix
