# Supplementary material for: Calcineurin Inhibitors Synergize with Manogepix to Kill Diverse Human Fungal Pathogens
Source: J Fungi (Basel). 2022 Oct 19;8(10):1102. doi: 10.3390/jof8101102 (PMC9605145; doi:10.3390/jof8101102)
Supplement: Supplementary file 1 [file jof-08-01102-s001.zip › jof-1967155-supplementary.pdf]

Supplemental figures

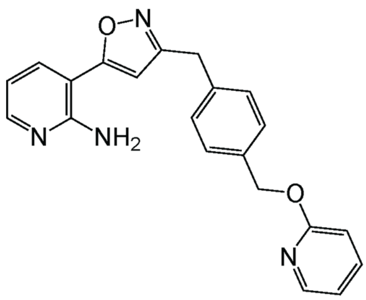

MGX

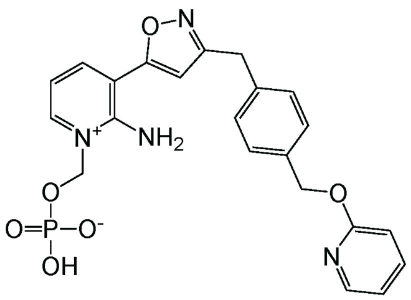

FMGX

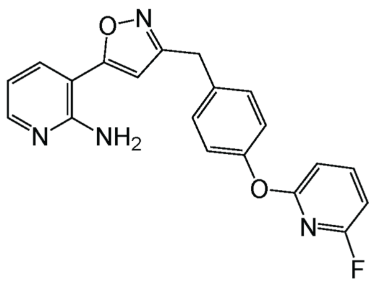

APX2039

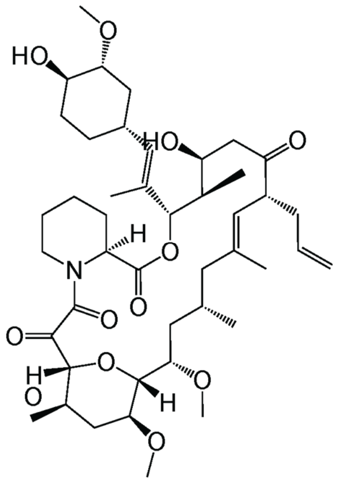

FK506

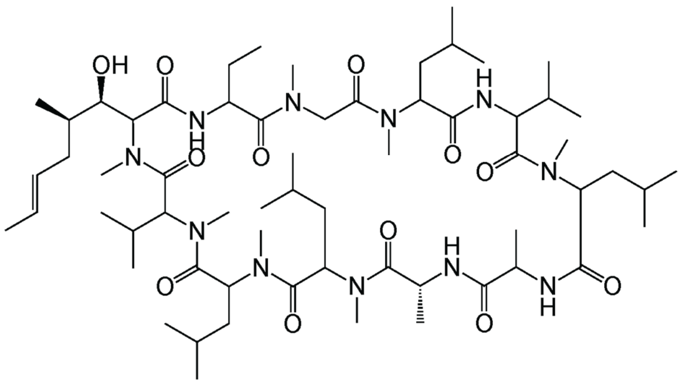

CsA

Figure S1. Chemical structures of relevant compounds

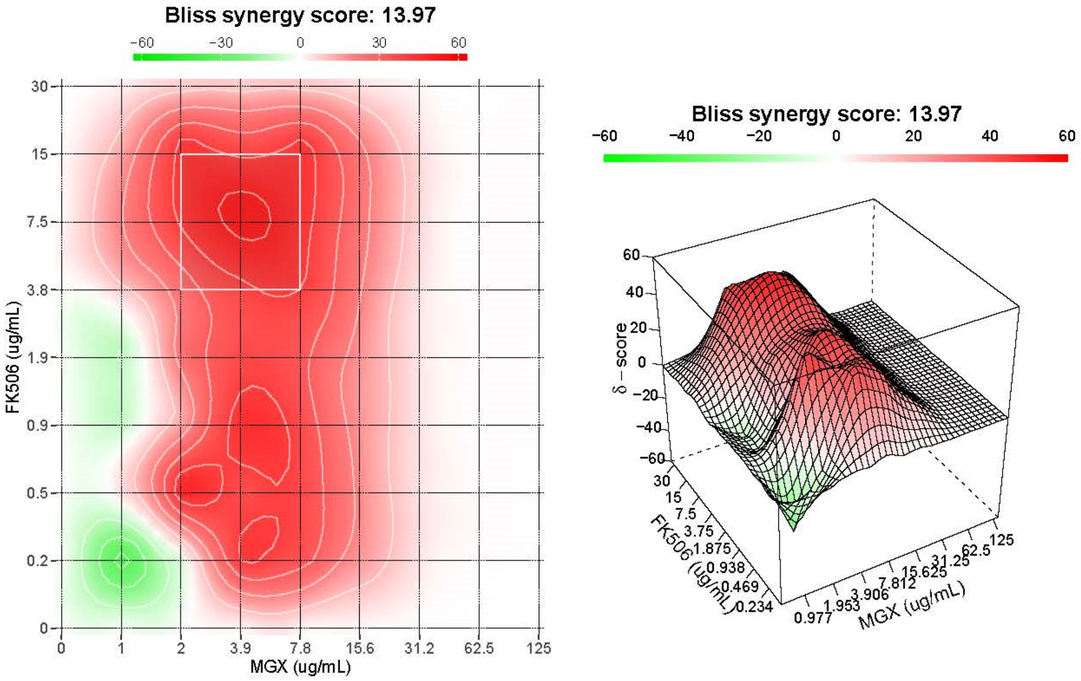

Figure S2. Bliss synergy analysis of antifungal interaction between MGX and FK506. *Growth data from the same dose response matrix of MGX and FK506 tested against* C. albicans *presented in Figure 1 were analyzed by Bliss model as implemented in the SynergyFinder web application version 3.0 (https://synergyfinder.fimm.fi/).*

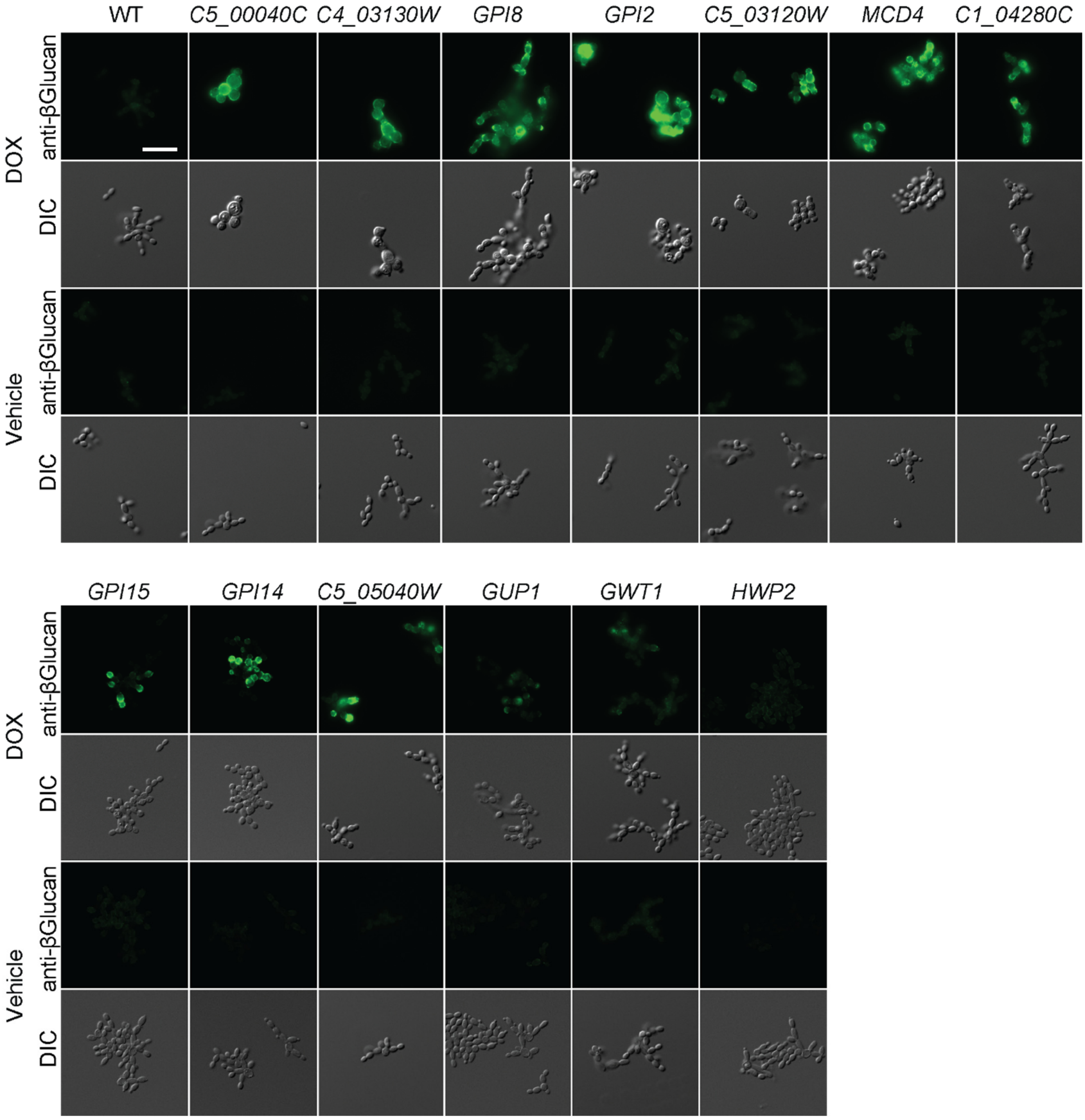

Figure S3. Transcriptional repression of GPI-anchor biosynthesis genes exposes (1→3)-β-D-glucan at the fungal cell surface. *Representative fluorescence micrographs of glucan-stained cells for each repressible mutant in the library showing > 2-fold change in median fluorescence intensity upon repression of the indicated gene in screening and validation flow cytometric experiments depicted in figure 5A. Conditional expression strains were grown in the presence or absence of DOX to repress target gene expression. Glucan staining and fluorescence microscopy were performed as in Figure 4. Scale bar, 20 µm.*

Table S1. GRACE strains used in this study. If no standard name was annotated in the Candida Genome Database, the standard name for the closest ortholog in *S. cerevisiae* is indicated in brackets. NE, non-essential; E – essential, GD, growth defect.

Feature Name	Standard Name	Annotation	Collection	Published Essentiality Score
C2_02900W_A	ERI1	1 - GlcNAc tranferase component	GRACE_v2	NE
CR_01430W_A	GPI15	1 - GlcNAc tranferase component	GRACE_v2	NE
C2_05280C_A	GPI19	1 - GlcNAc tranferase component	GRACE_v1	NE
C3_05390C_A	GPI1	1 - GlcNAc tranferase component	GRACE_v1	NE
CR_04040C_A	SPT14	1 - GlcNAc tranferase component	GRACE_v1	NE
CR_04470W_A	GPI2	1 - GlcNAc tranferase component	GRACE_v1	E
C1_10320W_A	(GPI12)	2- GlcNAc-PI de-N-acetylase	GRACE_v1	NE
C3_04710W_A	ARV1	X - GPI lipid flippase	GRACE_v2	NE
C2_05730C_A	GWT1	3 - GlcN-PI acyltransferase	GRACE_v1	NE
C4_02100C_A	GPI14	4 - acylGlcN-PI mannosyltransferase	GRACE_v1	NE
C6_01370W_A	(PBN1)	4 - acylGlcN-PI mannosyltransferase interactor	GRACE_v1	NE
C1_04280C_A	(GPI18)	5 - acylGlcN-PI mannosyltransferase 2	GRACE_v1	NE
C1_12200W_A	MCD4	6 - acylGlcN-PI-Man2 pEtN transferase	GRACE_v1	NE
C5_05040W_A	(GPI10)	7 - acylGlcN-PI-Man2 pEtN mannosyltransferase 3	GRACE_v1	NE
C2_03070C_A	Smp3	8 - acylGlcN-PI-Man3 pEtN mannosyltransferase 4	GRACE_v2	NE
C2_03920C_A	GPI13	9 - acylGlcN-PI-Man4 pEtN tranferase	GRACE_v1	NE
C1_05070C_A	GPI7	10 - acylGlcN-PI-Man4 pEtN tranferase	GRACE_v2	NE
C4_02420C_A	(GPI11)	10 - acylGlcN-PI-Man4 pEtN tranferase interactor	GRACE_v1	NE
CR_08190W_A	(GPI17)	11 - transaminase	GRACE_v2	NE
C1_07460C_A	GPI8	11 - transaminase	GRACE_v1	GD
C4_03130W_A	(GPI16)	11 - transaminase	GRACE_v1	E
C5_00040C_A	(GAA1)	11 - transaminase	GRACE_v1	E
C5_03120W_A	(GAB1)	11 - transaminase	GRACE_v1	GD
C1_04190C_A	BST1	12 - GPI deacylase	GRACE_v2	NE
C5_03460C_A	(PER1)	13 - deacylaase	GRACE_v1	NE
C1_13540W_A	GUP1	14 - acyltransferase	GRACE_v1	NE
C4_07200C_A	(CDC1)	15 - pEtN hydrolase	GRACE_v1	NE
C3_00470W_A	(TED1)	16 - pEtN hydrolase	GRACE_v1	NE
CR_01290C_A	CWH43	17 - GPI Lipid remodelase	GRACE_v2	NE
C2_01360C_A	DCW1	18 – GPI transglycosidase	GRACE_v1	NE
C2_00520W_A	DFG5	18 – GPI transglycosidase	GRACE_v1	NE
CR_03630W_A	IFF3	Predicted N-terminal signal sequence and C-terminal GPI anchor sequence	not constructed	
CR_03880W_A	IFF9	Predicted N-terminal signal sequence and C-terminal GPI anchor sequence	not constructed	
C3_01740C_A		Predicted N-terminal signal sequence and C-terminal GPI anchor sequence	not constructed	
C7_02300W_A	YPS7	Predicted N-terminal signal sequence and C-terminal GPI anchor sequence	GRACE_v2	NE

C5_00710W_A	IFF8	Predicted N-terminal signal sequence and C-terminal GPI anchor sequence	This Study	NE
C4_04050C_A	RHD3	Predicted N-terminal signal sequence and C-terminal GPI anchor sequence	GRACE_v2	NE
C5_03900W_A	PGA56	Predicted N-terminal signal sequence and C-terminal GPI anchor sequence	not constructed	
C4_03520C_A	RBT1	Predicted N-terminal signal sequence and C-terminal GPI anchor sequence	GRACE_v2	NE
CR_04900C_A	PGA39	Predicted N-terminal signal sequence and C-terminal GPI anchor sequence	GRACE_v2	NE
C2_03720W_A	PGA16	Predicted N-terminal signal sequence and C-terminal GPI anchor sequence	GRACE_v2	NE
C1_02360C_A	PGA5	Predicted N-terminal signal sequence and C-terminal GPI anchor sequence	not constructed	
CR_02750C_A	PGA34	Predicted N-terminal signal sequence and C-terminal GPI anchor sequence	GRACE_v2	NE
C2_00080C_A	FAV3	Predicted N-terminal signal sequence and C-terminal GPI anchor sequence	GRACE_v2	NE
C5_00410W_A		Predicted N-terminal signal sequence and C-terminal GPI anchor sequence	GRACE_v2	NE
CR_02290W_A	PGA22	Predicted N-terminal signal sequence and C-terminal GPI anchor sequence	GRACE_v2	NE
C4_06920C_A	CSA2	Predicted N-terminal signal sequence and C-terminal GPI anchor sequence	not constructed	
C2_09130C_A	IFF6	Predicted N-terminal signal sequence and C-terminal GPI anchor sequence	GRACE_v2	NE
CR_10480W_A	PGA1	Predicted N-terminal signal sequence and C-terminal GPI anchor sequence	GRACE_v2	NE
C4_01350W_A		Predicted N-terminal signal sequence and C-terminal GPI anchor sequence	GRACE_v2	NE
C7_03290C_A	RBR3	Predicted N-terminal signal sequence and C-terminal GPI anchor sequence	GRACE_v2	NE
CR_07070C_A	ALS3	Predicted N-terminal signal sequence and C-terminal GPI anchor sequence	This study	NE
C4_01000C_A	PGA57	Predicted N-terminal signal sequence and C-terminal GPI anchor sequence	GRACE_v2	NE
C4_00120W_A	PGA7	Predicted N-terminal signal sequence and C-terminal GPI anchor sequence	GRACE_v2	NE
C4_00130W_A	RBT5	Predicted N-terminal signal sequence and C-terminal GPI anchor sequence	GRACE_v2	NE
C1_03190C_A	ECM33	Predicted N-terminal signal sequence and C-terminal GPI anchor sequence	GRACE_v2	NE
C4_02370C_A	PGA59	Predicted N-terminal signal sequence and C-terminal GPI anchor sequence	GRACE_v2	NE
C4_06260W_A	PGA43	Predicted N-terminal signal sequence and C-terminal GPI anchor sequence	GRACE_v2	NE
C6_00160W_A	PGA48	Predicted N-terminal signal sequence and C-terminal GPI anchor sequence	GRACE_v2	NE
CR_04440C_A	RBR1	Predicted N-terminal signal sequence and C-terminal GPI anchor sequence	GRACE_v2	NE

CR_08510W_A	PGA13	Predicted N-terminal signal sequence and C-terminal GPI anchor sequence	GRACE_v2	NE
C1_05760C_A	PGA26	Predicted N-terminal signal sequence and C-terminal GPI anchor sequence	GRACE_v2	NE
C1_05960W_A	PGA45	Predicted N-terminal signal sequence and C-terminal GPI anchor sequence	GRACE_v2	NE
C1_13290W_A		Predicted N-terminal signal sequence and C-terminal GPI anchor sequence	GRACE_v2	NE
C2_00660C_A	SOD4	Predicted N-terminal signal sequence and C-terminal GPI anchor sequence	GRACE_v2	NE
C2_00800C_A	PGA27	Predicted N-terminal signal sequence and C-terminal GPI anchor sequence	GRACE_v2	NE
C2_00910W_A	PGA19	Predicted N-terminal signal sequence and C-terminal GPI anchor sequence	GRACE_v2	NE
C2_03350W_A	PGA17	Predicted N-terminal signal sequence and C-terminal GPI anchor sequence	GRACE_v2	NE
C2_03440W_A	PGA33	Predicted N-terminal signal sequence and C-terminal GPI anchor sequence	GRACE_v2	NE
C2_08430C_A	PGA46	Predicted N-terminal signal sequence and C-terminal GPI anchor sequence	GRACE_v2	NE
C2_08590W_A	YWP1	Predicted N-terminal signal sequence and C-terminal GPI anchor sequence	GRACE_v2	NE
C2_08980C_A	PGA55	Predicted N-terminal signal sequence and C-terminal GPI anchor sequence	GRACE_v2	NE
C2_09210W_A		Predicted N-terminal signal sequence and C-terminal GPI anchor sequence	GRACE_v2	NE
C3_01370C_A	PGA44	Predicted N-terminal signal sequence and C-terminal GPI anchor sequence	GRACE_v2	NE
C3_02300W_A	FGR23	Predicted N-terminal signal sequence and C-terminal GPI anchor sequence	GRACE_v2	NE
C3_03050C_A	PGA18	Predicted N-terminal signal sequence and C-terminal GPI anchor sequence	GRACE_v2	NE
C3_06190C_A	ALS6	Predicted N-terminal signal sequence and C-terminal GPI anchor sequence	GRACE_v2	NE
C3_07160W_A	PGA32	Predicted N-terminal signal sequence and C-terminal GPI anchor sequence	GRACE_v2	NE
C4_00450C_A	PGA10	Predicted N-terminal signal sequence and C-terminal GPI anchor sequence	This study	NE
C4_01330W_A		Predicted N-terminal signal sequence and C-terminal GPI anchor sequence	GRACE_v2	NE
C4_01340W_A		Predicted N-terminal signal sequence and C-terminal GPI anchor sequence	GRACE_v2	NE
C4_01360W_A	PGA53	Predicted N-terminal signal sequence and C-terminal GPI anchor sequence	GRACE_v2	NE
C4_02390W_A	PGA62	Predicted N-terminal signal sequence and C-terminal GPI anchor sequence	GRACE_v2	NE
C4_02440C_A	PGA38	Predicted N-terminal signal sequence and C-terminal GPI anchor sequence	GRACE_v2	NE
C4_03480C_A		Predicted N-terminal signal sequence and C-terminal GPI anchor sequence	This study	NE

C4_03510C_A	HWP2	Predicted N-terminal signal sequence and C-terminal GPI anchor sequence	GRACE_v2	NE
C4_04070C_A	PGA30	Predicted N-terminal signal sequence and C-terminal GPI anchor sequence	GRACE_v2	NE
C4_04470W_A	SAP10	Predicted N-terminal signal sequence and C-terminal GPI anchor sequence	GRACE_v2	NE
C4_04530C_A	PHR1	Predicted N-terminal signal sequence and C-terminal GPI anchor sequence	GRACE_v2	NE
C4_06310C_A	PGA41	Predicted N-terminal signal sequence and C-terminal GPI anchor sequence	GRACE_v2	NE
C4_06550C_A	IFF5	Predicted N-terminal signal sequence and C-terminal GPI anchor sequence	GRACE_v2	NE
C4_06560W_A	PGA15	Predicted N-terminal signal sequence and C-terminal GPI anchor sequence	GRACE_v2	NE
C6_01680C_A		Predicted N-terminal signal sequence and C-terminal GPI anchor sequence	GRACE_v2	NE
C6_03060W_A	PGA60	Predicted N-terminal signal sequence and C-terminal GPI anchor sequence	GRACE_v2	NE
C6_03690W_A	ALS5	Predicted N-terminal signal sequence and C-terminal GPI anchor sequence	not constructed	
C6_03700W_A	ALS1	Predicted N-terminal signal sequence and C-terminal GPI anchor sequence	This study	NE
C6_03860C_A	PGA61	Predicted N-terminal signal sequence and C-terminal GPI anchor sequence	GRACE_v2	NE
C6_04130C_A	ALS4	Predicted N-terminal signal sequence and C-terminal GPI anchor sequence	GRACE_v2	NE
C7_00190W_A		Predicted N-terminal signal sequence and C-terminal GPI anchor sequence	GRACE_v2	NE
C7_00860W_A	SSR1	Predicted N-terminal signal sequence and C-terminal GPI anchor sequence	GRACE_v2	NE
C7_03110W_A	PGA28	Predicted N-terminal signal sequence and C-terminal GPI anchor sequence	GRACE_v2	NE
CR_04420C_A	RBR2	Predicted N-terminal signal sequence and C-terminal GPI anchor sequence	GRACE_v2	NE
CR_09690C_A	PLB3	Predicted N-terminal signal sequence and C-terminal GPI anchor sequence	GRACE_v2	NE
C1_13450W_A	HYR1	Predicted N-terminal signal sequence and C-terminal GPI anchor sequence	GRACE_v2	NE
C2_08990C_A		Predicted N-terminal signal sequence and C-terminal GPI anchor sequence	GRACE_v2	NE
C3_06320W_A	ALS7	Predicted N-terminal signal sequence and C-terminal GPI anchor sequence	GRACE_v2	NE
C5_00730W_A	HYR3	Predicted N-terminal signal sequence and C-terminal GPI anchor sequence	GRACE_v2	NE
C6_00070C_A	PGA25	Predicted N-terminal signal sequence and C-terminal GPI anchor sequence	GRACE_v2	NE
C6_03710W_A	ALS9	Predicted N-terminal signal sequence and C-terminal GPI anchor sequence	GRACE_v2	NE
C7_00090C_A	CSA1	Predicted N-terminal signal sequence and C-terminal GPI anchor sequence	GRACE_v2	NE

CR_00610W_A	IFF4	Predicted N-terminal signal sequence and C-terminal GPI anchor sequence	GRACE_v2	NE
C1_00220W_A	PHR2	Predicted N-terminal signal sequence and C-terminal GPI anchor sequence	GRACE_v1	NE
C1_02630C_A	EXG2	Predicted N-terminal signal sequence and C-terminal GPI anchor sequence	GRACE_v1	NE
C1_08230C_A	PLB5	Predicted N-terminal signal sequence and C-terminal GPI anchor sequence	GRACE_v1	NE
C2_00100C_A	PGA52	Predicted N-terminal signal sequence and C-terminal GPI anchor sequence	GRACE_v1	NE
C2_00680C_A	SOD5	Predicted N-terminal signal sequence and C-terminal GPI anchor sequence	GRACE_v1	NE
C2_01380W_A	PLB4.5	Predicted N-terminal signal sequence and C-terminal GPI anchor sequence	GRACE_v1	NE
C3_01730C_A	UTR2	Predicted N-terminal signal sequence and C-terminal GPI anchor sequence	GRACE_v1	NE
C3_03870C_A	SAP9	Predicted N-terminal signal sequence and C-terminal GPI anchor sequence	GRACE_v1	NE
C3_04200W_A	AFP99	Predicted N-terminal signal sequence and C-terminal GPI anchor sequence	GRACE_v1	NE
C4_02900C_A	CRH11	Predicted N-terminal signal sequence and C-terminal GPI anchor sequence	GRACE_v1	NE
C5_00270W_A	PGA14	Predicted N-terminal signal sequence and C-terminal GPI anchor sequence	GRACE_v1	NE
C5_02460C_A	ECM331	Predicted N-terminal signal sequence and C-terminal GPI anchor sequence	GRACE_v1	NE
C5_03050C_A	PGA58	Predicted N-terminal signal sequence and C-terminal GPI anchor sequence	GRACE_v1	NE
C5_04130C_A	CHT2	Predicted N-terminal signal sequence and C-terminal GPI anchor sequence	GRACE_v1	NE
C5_04370C_A	PGA37	Predicted N-terminal signal sequence and C-terminal GPI anchor sequence	GRACE_v1	NE
C5_04800W_A	CRH12	Predicted N-terminal signal sequence and C-terminal GPI anchor sequence	GRACE_v1	NE
C5_05390C_A	PGA4	Predicted N-terminal signal sequence and C-terminal GPI anchor sequence	GRACE_v1	NE
C6_03850C_A	IHD1	Predicted N-terminal signal sequence and C-terminal GPI anchor sequence	GRACE_v1	NE
CR_00180C_A	CHT1	Predicted N-terminal signal sequence and C-terminal GPI anchor sequence	GRACE_v1	NE
CR_02280W_A	PGA23	Predicted N-terminal signal sequence and C-terminal GPI anchor sequence	GRACE_v1	NE

Table S2. Supplementary population statistics for Figure 5A.

Tube Name:	Abort(%):	All Events	P1 Events	P1 % Total	All Events Median FSC-A	P1 Median FSC-A	All Events Median SSC-A	P1 Median SSC-A	All Events Mean FITC-A	P1 Mean FITC-A	All Events Median FITC-A	P1 Median FITC-A
WT - NoDOX	8.48	30000	28443	94.81%	534375.9	552420.8	136679	133481.3	43761.8	33572.4	15965.9	16326.4
WT - DOX	10.5	30000	29525	98.42%	635350.3	642884	144233.3	143980.5	38652.6	37510.1	16754.9	16974.2
C5_00040C - NoDOX	7.69	30000	29757	99.19%	675746.8	681026.1	149110.2	148137.3	47969	48118.6	21314.8	21456.2
C5_00040C - DOX	0.5	30000	25464	84.88%	1291591	1432379	665506.8	757493.9	1473131	1617788	1054182	1199509
C4_03130W - NoDOX	10.1	30000	29791	99.30%	548295.3	549061.3	120523.6	120049.7	37616.2	37441.6	16523.4	16596.7
C4_03130W - DOX	1.07	30000	27251	90.84%	1370945	1466335	623421.9	667595.1	1183601	1256783	779337.6	847709.1
GPI8 - NoDOX	8.53	30000	29742	99.14%	595287	597842	128027.2	127345.6	40665	40378.2	18069.8	18175.6
GPI8 - DOX	1.27	30000	27705	92.35%	1574478	1692241	596964.6	650039.1	1114359	1159703	680779.9	737598.7
GPI2 - NoDOX	7.59	30000	29562	98.54%	588634.3	593042.6	130154.3	129123.3	46104.8	45907.1	20436.8	20649.3
GPI2 - DOX	0.91	30000	26550	88.50%	1204370	1325960	527886.6	582573.1	969460.3	1056632	574969.8	662706.4
C5_03120W - NoDOX	6.65	30000	29721	99.07%	546496.3	548951.6	121602.8	121172.7	35384.9	35047.5	16263.4	16387
C5_03120W - DOX	2.16	30000	28713	95.71%	1157202	1182821	403836.8	410154.3	487191.6	499995.7	364126.8	378590.4
MCD4 - NoDOX	9.28	30000	29812	99.37%	537793.4	539161.8	114797.5	114457.8	40185.1	39018.1	16274.5	16315
MCD4 - DOX	3.18	30000	29186	97.29%	1367181	1381647	407612.7	411160.3	382904.5	389597.8	292064.1	298477.7
C1_04280C - NoDOX	9.28	30000	29769	99.23%	546866.1	548339.6	121392.1	121185.1	32672	32290.8	14841.8	14906.2
C1_04280C - DOX	4.32	30000	29435	98.12%	1481343	1494561	419273.9	422285.5	277156.3	279193	197319.5	202256.7
GPI15 - NoDOX	3.76	30000	29263	97.54%	545271.6	556940.9	122521.9	120376.2	62404.2	62706.1	31204.8	31945.3
GPI15 - DOX	4.61	30000	29127	97.09%	1344290	1371899	347475.2	351084.9	263081.5	268240.5	111024.8	117566.3
GPI14 - NoDOX	6.11	30000	29575	98.58%	597694.9	603014.7	134404.2	132520.6	50731.2	50366.4	23808.8	24103.7
GPI14 - DOX	3.82	30000	29340	97.80%	1222969	1237620	282874.6	284188.8	197822.3	200342.2	76778.1	79144.8
C5_05040W - NoDOX	8.99	30000	29774	99.25%	569577.5	571150.4	127915.9	127609.7	47334.6	43791.5	17911.6	17989.4
C5_05040W - DOX	5.14	30000	29490	98.30%	1344546	1356890	334066.3	334853.6	166600.1	165998.8	72916.8	74480
GUP1 - NoDOX	6.1	30000	29672	98.91%	556107.1	560711.3	123446	122534.8	51796.5	51417.7	22961.5	23167.4
GUP1 - DOX	7.68	30000	29776	99.25%	1135466	1140376	285012.8	284958.5	115318.2	115658.8	66181.1	66783.8
GWT1 - NoDOX	10.1	30000	29863	99.54%	561072.1	561938.1	127714	127441.6	29735.7	29191.2	13706.6	13733.8
GWT1 - DOX	6.83	30000	29625	98.75%	1235861	1244711	295943.3	295998.9	111628.1	111255.5	46731.7	47343.4

HWP2 - NoDOX	5.9	30000	29709	99.03%	538163.3	541467	115750.2	114971.2	24146.5	23083.9	6687	6712.8
HWP2 - DOX	6.47	30000	29730	99.10%	562398.5	565436.8	124351.3	123765.5	43385	41472.9	18707.3	18829.2

Table S3. Supplementary population statistics for Figure 5B

Tube Name:	Abort(%):	All Events	P1 Events	P1 % Total	All Events Median FSC-A	P1 Median FSC-A	All Events Median SSC-A	P1 Median SSC-A	All Events Mean PB450-A	P1 Mean PB450-A	All Events Median PB450-A	P1 Median PB450-A
WT - NoDOX	8.59	30000	29637	98.79%	532514.6	536042.6	119047.5	118730.8	378.2	348.2	185.2	183.9
WT - DOX	9.86	30000	29411	98.04%	637569.8	647532.6	146272.3	145256	456.4	412.4	218.3	214
GAA1 - NoDOX	7.38	30000	29634	98.78%	705490.9	715675.9	154154.7	153409.3	600.9	589.7	282.8	281.2
GAA1 - DOX	0.45	30000	23863	79.54%	1264690	1485980	685821.3	838417.7	8783.4	10620.1	5802.5	8049.7
C4_03130W (GPI16) - NoDOX	8.84	30000	29650	98.83%	564107.5	568615.5	123174.8	122839.5	446.6	430.6	210.4	209.4
C4_03130W (GPI16) - DOX	0.9	30000	26391	87.97%	1402055	1540509	671593.9	745269.7	7322.3	8042.1	5130.9	6009.2
GPI2 - NoDOX	8.04	30000	29326	97.75%	596520.1	608322.8	134265	133194.8	512.1	484.4	251.5	246
GPI2 - DOX	0.81	30000	25063	83.54%	1178420	1361857	543561.1	637643.8	7271.7	8384.4	4662.7	6157.9
GPI8 - NoDOX	8.75	30000	29598	98.66%	604497.8	609150.5	129034	129167.8	557.8	522.6	246	245.9
GPI8 - DOX	1.19	30000	27004	90.01%	1733899	1920164	723494.9	815005.8	9416.8	9869.8	5043.1	5973
C5_03120W (GAB1) - NoDOX	7.06	30000	29538	98.46%	640563.4	648287.9	145248.7	144837.8	510.6	482.2	243.6	240.9
C5_03120W (GAB1) - DOX	1.62	30000	27987	93.29%	1167451	1213621	427110.6	441808.4	3103.4	3243	1912.5	2033.4
MCD4 - NoDOX	9.36	30000	29716	99.05%	573819	576647.3	121338.8	121039.8	519.4	494.8	237.8	237.4
MCD4 - DOX	3.96	30000	29259	97.53%	1372362	1387108	424445.3	427874	2010.1	2005.2	1513.1	1534.6
C4_02420C (GPI11) - NoDOX	6.76	30000	29630	98.77%	569909.6	573661.2	122008.3	121623.3	477.3	435.4	228.3	226.9
C4_02420C (GPI11) - NoDOX	3.04	30000	29011	96.70%	1697351	1728763	484227.6	490856.6	1839.5	1822.8	1424.9	1455.7
GPI18 - NoDOX	9.29	30000	29680	98.93%	564054.5	566647.4	124234.2	124030.6	447.5	397.5	206.2	205.1
GPI18 - DOX	4.03	30000	29096	96.99%	1480103	1500720	427498.4	431087.4	1820.5	1776.1	1327.9	1348.2
GWT1 - NoDOX	10.82	30000	29772	99.24%	596179.9	598235.8	132745.4	132568.9	449.5	422	201.4	200.7
GWT1 - DOX	6.99	30000	29638	98.79%	1308825	1323266	318998.4	321335.5	1211.3	1195.7	751	756.7
GPI15 - NoDOX	3.29	30000	28826	96.09%	564617.4	588812.8	155581	154440	463.5	413.4	242.9	235.9
GPI15 - DOX	4.06	30000	28522	95.07%	1371095	1426337	361792.8	372796.5	1082.2	1071.2	750.8	773
GUP1 - NoDOX	7.05	30000	29620	98.73%	591584.1	597503.1	168225.8	167998.3	412.6	385	215	213.5
GUP1 - DOX	7.06	30000	29696	98.99%	1205815	1212389	307488.8	308474.1	868.5	849.2	604.3	607.2
